# Primary Pulmonary Hypoplasia

**DOI:** 10.5334/jbsr.1717

**Published:** 2019-01-15

**Authors:** Jeremy Liners, Laurent Médart, Laurent Collignon

**Affiliations:** 1 Department of Radiology, CHR Liège, BE

**Keywords:** Bronchopulmonary malformations, hemithorax whiteout, computed tomography

## Case Report

**Figure 1:**
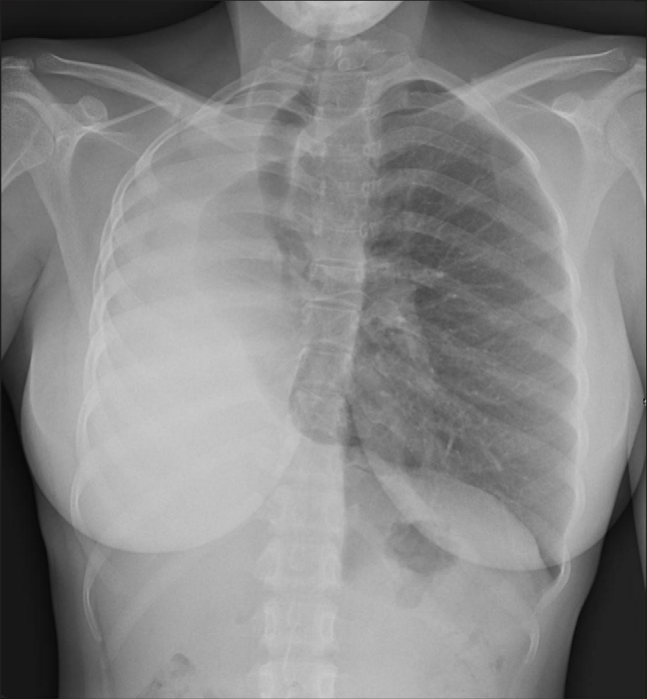


**Figure 2:**
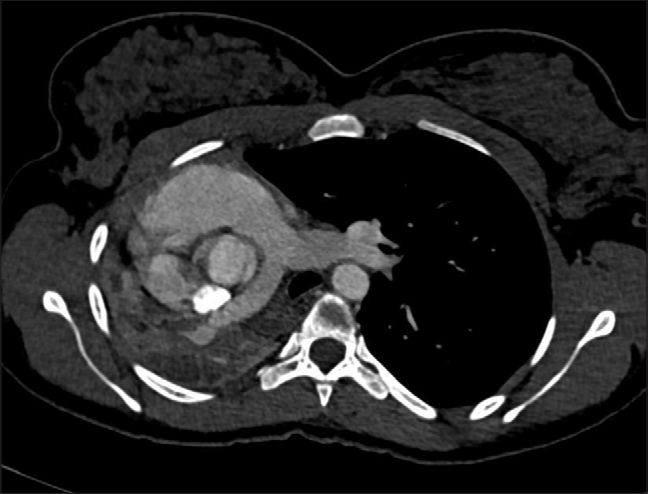


**Figure 3:**
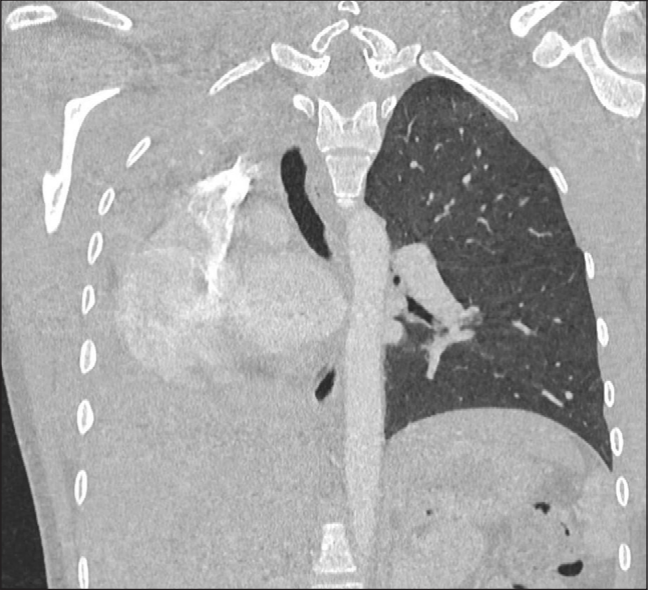


An 18-year-old woman of African origin presented to the emergency room for progressive stress dyspnea and expiratory wheezing for one year, worsening for six months. It was associated with thoracic tightness and productive cough, without hemoptysis or fever. Perinatal history was unremarkable, and no history of similar symptoms was found in any of her siblings. Neither ongoing treatment nor smoking habits were reported. Clinical exam revealed decreased breath sounds to the right pulmonary base with dullness to percussion and diminished vocal fremitus on the right side. Pulse oximetry was 100%, and neither tachycardia nor tachypnea were noticed. Plain chest radiography showed absence of inflated right lung with mediastinal shift towards the same side (Figure 1). Contrast-enhanced chest computed tomography (CT) demonstrated complete atelectasis of the right lung with short-blind right main bronchus and compensatory hyperinflation of the left lung; proposed diagnosis was congenital bronchopulmonary malformation (pulmonary hypoplasia) (Figures 2 and 3). Lung function tests confirmed restrictive lung disease, with FEV reaching 40% of the predicted value and FVC 43%. Bronchoscopy revealed a fibrotic scar area on the right side of the trachea with dimple at the place of opening of right main bronchus.

## Discussion

Pulmonary hypoplasia is one of the many congenital bronchopulmonary malformations characterized by pulmonary underdevelopment [1]. These abnormalities have been classified by Schneider in three major types according to the developmental stage of the primitive pulmonary bud, then modified by Boyden as follows:

- Type 1 (agenesis): no trace of lung, bronchus or vascular supply on the affected side.
- Type 2 (aplasia): rudimentary bronchus without any pulmonary tissue.
- Type 3 (hypoplasia): presence of variable amounts of bronchial tree, pulmonary parenchyma, and supporting vasculature.

Incidence of pulmonary hypoplasia ranges from 9 to 11 per 10,000 live births. It generally presents during neonatal or early childhood period rather than adult period which is rare. It can be categorized in two forms:

- primary, related to an embryologic defect or an in utero accident, and
- secondary, associated to conditions that limit fetal lung growth.

Clinical presentation varies depending of the severity of pulmonary and vascular anomalies. Some patients can remain asymptomatic all their life. Nevertheless, most of them present with repeated respiratory infections and wheezing, due to underdevelopment of the lung, with decrease in vital capacity.

Plain radiography shows absence of aeration of the affected lung, with ipsilateral mediastinal shift. CT is the most effective noninvasive modality to study pulmonary vasculature and bronchial tree. Bronchoscopy is also useful to demonstrate rudimentary bronchus.

No treatment is required in asymptomatic cases. For others, management consists to relieve respiratory complaints by B2 mimetics and to treat recurrent respiratory infections by antibiotics and inhaled corticosteroids. In case of too many recurrent infections, pneumectomy can be discussed.

Unilateral primary pulmonary hypoplasia is a very rare discovery in adulthood. This entity should be considered in the differential diagnosis for hemithorax whiteout on chest radiography, especially in case of history of recurring respiratory infections.
